# Adaptive Feedforward Compensating Self-Sensing Method for Active Flutter Suppression

**DOI:** 10.3390/s18103447

**Published:** 2018-10-13

**Authors:** Yizhe Wang, Zhiwei Xu

**Affiliations:** State Key Laboratory of Mechanics and Control of Mechanical Structures, Nanjing University of Aeronautics and Astronautics, Nanjing 211106, China; zhwxu@nuaa.edu.cn

**Keywords:** self-sensing actuation, feedforward compensation, capacitance mismatching, local strain, steepest descent algorithm, active flutter suppression

## Abstract

A single piezoelectric patch can be used as both a sensor and an actuator by means of the self-sensing piezoelectric actuator, and the function of self-sensing shows several advantages in many application fields. However, some problems exist in practical application. First, a capacitance bridge circuit is set up to realize the function of self-sensing, but the precise matching of the capacitance of the bridge circuit is hard to obtain due to the standardization of electric components and variations of environmental conditions. Second, a local strain is induced by the self-sensing actuator that is not related to the global vibration of the structure, which would affect the performance of applications, especially in active vibration control. The above problems can be tackled by the feedforward compensation method that is proposed in this paper. A configured piezoelectric self-sensing circuit is improved by a feedforward compensation tunnel, and a gain of compensation voltage is adjusted by the time domain and frequency domain’s steepest descent algorithms to alleviate the capacitance mismatching and local strain problems. The effectiveness of the method is verified in the experiment of the active vibration control in a wind tunnel, and the control performance of compensated self-sensing actuation is compared to the performance with capacitance mismatching and local strain. It is found that the above problems have negative effects on the stability and performance of the vibration control and can be significantly eliminated by the proposed method.

## 1. Introduction

Self-sensing piezoelectric actuators are used as sensors and actuators simultaneously in active vibration control, mass detection, and condition monitoring. They have many advantages over separate piezoelectric sensors and actuators, such as guaranteed system stability and the prevention of spillover. The self-sensing actuators separate the driving voltage and measurement signal by the bridge circuit that was first proposed by Dosch [1]. Since the measurement and actuator piezoelectric patch (lead zirconate titanate, PZT) are collocated, it is lighter and costs less than single piezoelectric sensors and actuators. Furthermore, a self-sensing actuator applies the driven force to the same location of the sensor, which avoids the possibility of instability caused by the location of the sensor and actuator on the modal node.

The application of self-sensing actuators has been widely studied in the past. Self-sensing PZT actuators have been widely employed in vibration and acoustic control, condition monitoring, and energy harvesting [2,3,4,5]. Tzou et al. studied the vibration control of an Euler–Bernoulli beam using self-sensing spatial orthogonal actuators [6]. Frampton et al. studied the use of self-sensing actuators for active flutter suppression [7]. Bo proposed a novel self-sensing vibration control method and applied a self-sensing electromagnetic transducer to suppress the vibration of a space antenna reflector [8]. Hu proposed a general circuit topology of enhanced indirect-driven self-sensing actuators to improve the rejection capability of audio-induced vibration [9]. Furthermore, self-sensing actuators were also applied in the field of active acoustic control [10,11] and health monitoring [12,13,14]. Qiu et al. applied the self-sensing actuator concept to PZT stack actuators used as a micropositioner [15]. The authors used a nonlinear element in the self-sensing circuit to negate any nonlinear effects, thus improving the signal-to-noise ratio. Sodano et al. studied the use of macrofiber composites (MFCs) in self-sensing actuators for the alleviation of vibrations of inflatable structures [16]. Samira proposed a method to detect adsorbed masses by self-sensing piezoelectric microsensors, which improved the platform with higher sensitivity and selectivity for the detection of smaller masses [17].

The advantages of self-sensing actuators are remarkable according to the published literature; however, they also turn out to have a major defect in origin configuration, which limits their practical and commercial use. The essence of self–sensing actuators is the balance of the bridge circuit, but this is hard to implement. The match capacity of the PZT is hard to obtain due to the standard specifications in production and sensitivity to environmental temperature [18,19,20]. When used as an actuator, the voltage applied to the PZT material tends to be 100 times greater than the voltage generated during sensing. Since sensing and driving signals are applied simultaneously on the piezoelectric patch, it is hard to separate the sensing voltage from the mixed signals. The PZT that is used in self-sensing actuators can lead to control stability problems if the environmental conditions are not matched to the properties of the PZT materials [21]. 

To overcome the defect of the self-sensing bridge, some improved methods have become a topic of research; the current study is concerned with the development of robust and adaptive self-sensing actuators. Cole et al. proposed an adaptive filter algorithm to measure the capacitance of the piezoelectric material to monitor the parametric variations of self-sensing circuits [22,23]. Ye proposed a new adaptive signal separation method based on the bridge circuit, which can separate a relatively small sensing voltage from related mixed voltage adaptively [24]. Many researchers have cast the adaptive self-sensing actuator in the context of state-space observer-based problems. Dong utilized the observer for self-sensing with an improved circuit that involves the voltage-driving and capacitance-measuring functions [25]. Okugawa constructed a self-sensing system by an identification and state-space estimation method to increase the control stability [26]. All of these methods behave in the implementation of software, where the measurement signal can be accurately estimated within a certain range; however, the actual measurement signal is affected by the driving signal. When the bridge is unbalanced, it will cause the measurement signal to exceed the allowable range of the measuring circuit and even break down the circuit. Nishigaki used two piezoelectric materials with equivalent capacitance to compensate for changes in temperature [27]. Simmers evaluated the effects of an unbalanced bridge circuit and quantified the variations in PZT capacitance as a result of temperature changes [28]. The new design included adding a capacitor in a series or parallel to the PZT and matched capacitance. It was found that the added capacitors increased the system stability with a slight decrease in the effectiveness of the vibration reduction. This method can reduce the influence of the bridge imbalance to the vibration control under certain conditions, but it cannot completely eliminate the interference in the measurement, and it will consume more driving energy. Time multiplexed configuration is another way to avoid the bridge imbalance. Time division self-sensing actuators were proposed by Wang and Wang [29], where the piezoelectric patch is connected with a sensor and actuator circuit in turns through the shift of a switch controlled by a time base signal. Suresh et al. proposed a simple circuit for a piezoelectric actuator that used a potential dividing concept for resonant mass sensors [30]. Later, an analog self-sensing circuit was proposed, in which the half-periods of a sinusoidal signal were used for actuator and sensing [31]. Although time division multiplexing can accurately measure the piezoelectric induction signal, it makes the excitation discontinuous, so that it is not applicable in the field of vibration control. Ruppert used charge amplifier-based self-sensing configurations that did not suffer from a capacitive circuit mismatch and were insensitive to parasitic capacitance on the operation amplifier input [32,33]. Putra designed an adaptive controller to compensate for the nonlinearity and output disturbance of the self-sensing circuit [34,35].

With the exception of capacitance mismatching, piezoelectric actuation is also confronted with the problem of inducing local strain in the structure to which it is coupled. The local strain-induced signal is measured by the piezoelectric patch, which is typically not related to the vibration of the global structure, and in general has a negative effective on the stability and performance of active vibration control. To overcome the local strain problem, Ji and Wu investigated the influence of local strain by a numerical and experimental method and separated the local strain signal by using neural network identification [36]. Michau observed the bending nearfield problem in active acoustic control by self-sensing actuation and proposed a method to compensate for the transfer function of the modified model [37]. Pelletier proposed a digital compensation method for the signal induced by local strain based on the PZT and cantilever beam parameters [38].

In this study, an adaptive feedforward compensation method is proposed that can treat the problems of capacitance mismatching and local sensing in a unified manner. An improved configuration of a piezoelectric self-sensing circuit is designed that is subject to the imbalance of the bridge, and does not affect the excitation voltage applied to the PZT. The improved self-sensing piezoelectric circuit compensates the measurement signal by a feedforward tunnel in which the gain is adjusted by a digital potentiometer. A time domain steepest descent (TDSD) method is first implemented for the problem of capacitance mismatching by adjusting the gain of the feedforward voltage, then the frequency domain steepest descent (FDSD) method is implemented to further modify the compensation gain to alleviate the local strain for vibration control. The new self-sensing circuit is utilized in the flutter suppression of flexible wings in a wind tunnel that exhibits effectiveness in control stability. 

## 2. Self-Sensing Piezoelectric Actuator

### 2.1. Original Self-Sensing Piezoelectric Actuator

**Figure 1:**
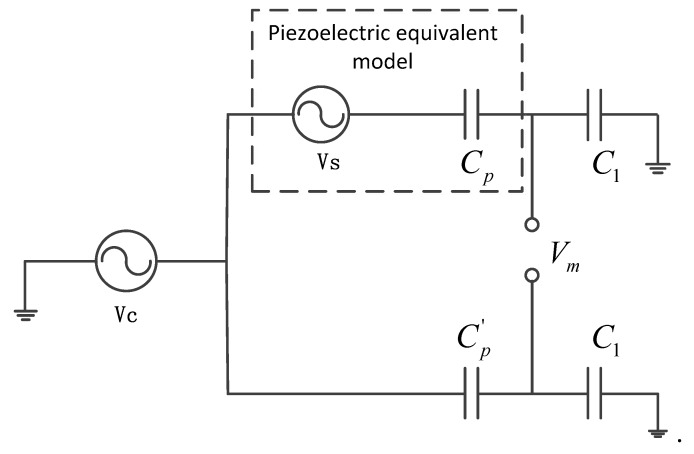
Equivalent circuit of original self-sensing piezoelectric actuation.

The original piezoelectric self-sensing actuator bridge is shown in Figure 1. An equivalent electric model of the piezoelectric element is composed of a voltage source $V_{s}$ in series with capacitance $C_{p}$. The voltage source accounts for the strain sensing and the capacitor accounts for the dielectric properties of the piezoelectric. The matching capacitor $C_{p}^{'}$ and capacitor $C_{1}$ are used to construct the bridge circuit, which subtracts the driving voltage from the measurement signal to obtain the sensing signal.

The constitutive equations of piezoelectric materials involve the variables of stress, strain, electric field, and electric displacement, which are represented by a single pair of electromechanical equations:

(1) $$\begin{array}{l} \varepsilon =s^{E}\sigma +d_{t}E\\ D=d\sigma +\varepsilon^{T}E \end{array}$$

where ε,σ,D,E are the strain, stress, electric displacement, and electric field, respectively, and $s^{E},d,\varepsilon^{T}$ denote the elastic compliance and piezoelectric constant. When the driving voltage and induced voltage are applied to the piezoelectric material simultaneously, the measurement voltage can be expressed by the integration of the whole piezoelectric field Ω:

(2) $$V_{m}=C_{p}\underset{\Omega }{∭}\left(d\sigma +\varepsilon^{T}E\right)d\Omega \;$$

The measurement voltage $V_{m}$ between two poles of the PZT is composed of the control voltage $V_{c}$ and the sensing voltage $V_{s}$:

(3) $$V_{m}=V_{c}+V_{s}\;$$

In order to utilize a self-sensing PZT actuator, it is necessary to separate the control voltage $V_{c}$ supplied to the PZT for the actuator from the sensing voltage $V_{s}$ created by the material’s deformation. The separation of voltage is performed by a bridge circuit; the PZT can be modeled as a voltage source $V_{c}$ and capacitor $C_{p}$ in series, resulting in a bridge circuit that is balanced with a capacitor of matched capacitance $C_{p}^{'}$ as shown in Figure 1. The measurement voltage of the self-sensing bridge is given by the following equation:

(4) $$V_{m}=(\frac{C_{p}}{C_{1}+C_{p}}-\frac{C_{p}^{'}}{C_{1}+C_{p}^{'}})V_{c}+\frac{C_{p}}{C_{1}+C_{p}}V_{s}\;$$

It can be seen from the expression that the measurement output is equivalent to the voltage induced by the deformation of the piezoelectric patch, while the captaincy is matched, i.e., $C_{p}^{'}=C_{p}$, the self-sensing actuator, is in ideal working condition. The measurement voltage of bridge output is proportional to the sensing voltage:

(5) $$V_{m}=\frac{C_{p}}{C_{1}+C_{p}}V_{s}\;$$

**Figure 2:**
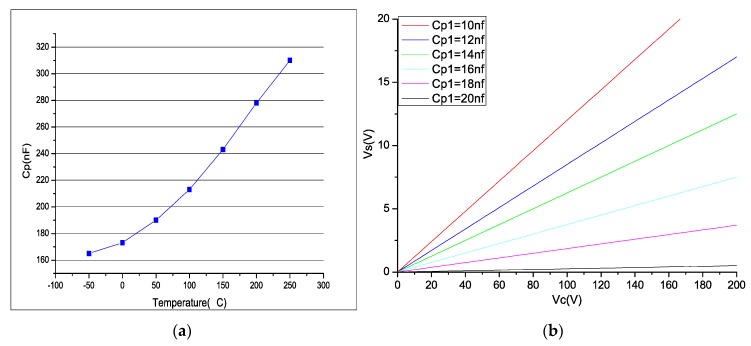
(**a**) Piezoelectric capacitance vs. temperature; (**b**) peak value curve of the driven and inference voltage.

Figure 2a shows the relationship with the capacitance and temperature of the PZT 5A material (70 × 10 × 0.1 mm³), which indicates the sensitivity to the temperature variation. Since the driving voltage is often several orders of magnitude larger than the induced voltage, even a small capacitance mismatch will have a greater impact on the output error. In this study, the capacitance of the MFC piezoelectric patch is 15.5 nF and $C_{1}$ = 50 nF. Figure 2b shows the variation curve of the measurement error voltage peak with the capacitance mismatch. 

It can be seen from the figure that even if the difference between the capacitance and actual capacitance of the piezoelectric material is small, a substantial interference voltage will be produced at the measuring end when the peak value of the driving voltage is large. The interference voltage is so great that is possible to exceed the allowable value of the measuring circuit, so that a bridge circuit based on capacitance matching is limited by the capacitance deviation. To suppress the interference caused by a capacitance mismatch, in this study, the original self-sensing piezoelectric bridge is improved.

### 2.2. Voltage Feedforward Compensation Self-Sensing Configuration

**Figure 3:**
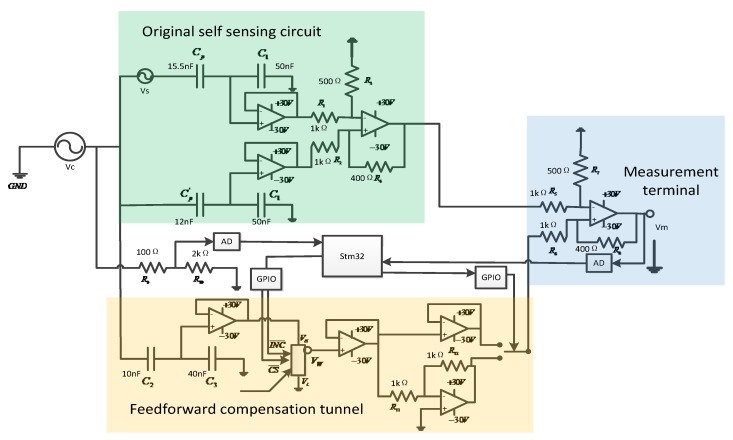
Improved self-sensing piezoelectric actuation circuit.

A self-sensing piezoelectric bridge based on an adaptive gain adjustment compensation method was designed that alleviates the disturbance of driven voltage from the mismatched capacitor by the feedforward voltage to get over the deflection of the original self-sensing piezoelectric bridge circuit. The voltage feedforward compensation configuration is shown in Figure 3.

As shown in Figure 3, a feedforward tunnel was added to the traditional capacitance matching bridge. The output voltage of the feedforward channel is proportional to the driving voltage, and the gain of the tunnel is regulated by the digital potentiometer. According to Equation (2), the disturbance signal caused by capacitance mismatch is also directly proportional to the driving voltage, so that there is an optimal gain to eliminate the error signal completely.

In the feedforward tunnel, the compensating signal is derived from the driving signal and applied to the digital potentiometer through the capacitor divider. The gain of the compensating voltage is modified by the digital potentiometer, which is applied to the measurement output by the voltage follower or subtraction circuit. The mismatch of capacitance can be divided into two different cases, as follows.

When the matching capacitance is less than the piezoelectric capacitance, the input signal is in the same phase as the driving voltage, and the output signal of the feedforward path is applied to the measured signal through the voltage follower by analog switching. When the matching capacitance is greater than the piezoelectric equivalent capacitance, the input signal is opposite to the driving voltage, and the output signal of the feedforward path is applied by the reverse amplification circuit.

The connection option is determined by the analog switch, and the measurement voltage of the output terminal of the bridge can be expressed as:

(6) $$V_{m}=(\frac{C_{p}}{C_{1}+C_{p}}-\frac{C_{p}^{'}}{C_{1}+C_{p}^{'}})V+\frac{C_{p}}{C_{1}+C_{p}}V_{s}\pm kV\;$$

Since the phase of compensation signal is identical to the driven voltage, feedforward interference can be completely compensated by the feedforward tunnel.

The self-sensing circuit is dependent on the ability of the feedforward voltage to compensate for the piezoelectric capacitance dynamically. For this, an adaptive estimation strategy for piezoelectric capacitance value is utilized. The adaptive estimation mechanism is a remedy for the automatic tuning of the capacitance bridge. Along this line, a technique to adaptively turn the digital potentiometer in real time is presented.

### 2.3. Adaptive Compensatory Mechanism for Capacitance Mechanism

**Figure 4:**
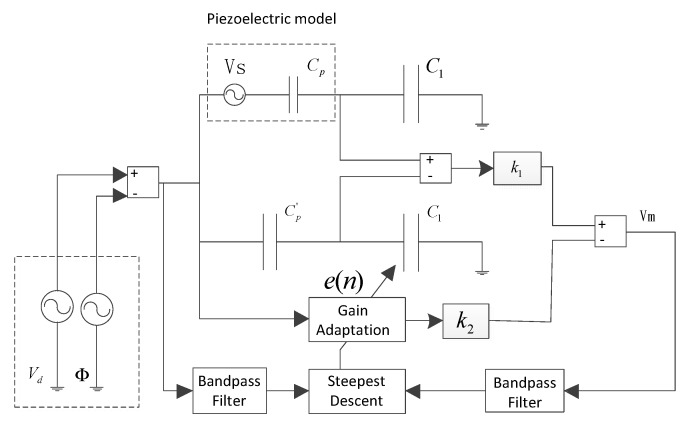
Schematic of the adaptive strategy for capacitance mismatching.

For practical implementation of self-sensing, the piezoelectric capacitance $C_{p}$ must be compensated in real time. The output voltage will reflect the sensing voltage only if the condition $C_{p}=C_{p}^{'}$ is satisfied. The adaptive compensatory mechanism that is shown in Figure 4 performs an online modification of the gain of compensation tunnel and uses an adaptive algorithm to dynamically drive the error to minimum. For this, a low-power persistent excitation signal Φ, which is at a different frequency from the control voltage $V_{d}$, is used as the test signal. The low power ensures that the voltage signals do not induce a moment in the piezoelectric actuator, and hence do not set the structure into vibration. Then, a bandpass filter is utilized to filter out the driven voltage in the process of adaptive iteration.

The adjustment of the digital potentiometers is realized by the steepest gradient method. The regulation formula of the steepest gradient method is given by:

(7) $$w(n+1)=w(n)-c\frac{\partial E}{\partial w(n)}\;$$

where w is the parameter to be adjusted, E is the objective function, and c is a constant that ensures iterative convergence. The steepest gradient method shows good performance for the objective function with a unique extreme value, so it is used for the adjustment of a digital potentiometer. The objective function is selected to be:

(8) $$E={\left(\frac{C_{p}}{C_{1}+C_{p}}k_{1}-\frac{C_{p}^{'}}{C_{1}+C_{p}^{'}}ek_{2}^{}\right)}^{2}V_{c}{}^{2}\;$$

where $k_{1}$ is the driving voltage interference gain derived from capacitance mismatch in the original self-sensing piezoelectric bridge, $k_{2}^{}$ is the gain of the driving voltage signal to the measuring end in the compensation tunnel, and e is the gain of the digital potentiometer as the dependent variable of the objective function. The digital potentiometer is utilized as a voltage divider in the compensation channel and changes the value between −1 and 1. The partial deviation is taken for the dependent variable of the objective function:

(9) $$\frac{\partial E}{\partial e}=2\frac{C_{p}^{'}}{C_{1}+C_{p}^{'}}k_{2}(ek_{2}-k_{1})V_{c}^{2}\;$$

Since $\frac{C_{p}^{'}}{C_{1}+C_{p}^{'}}(ek_{2}-k_{1})V_{c}$ is the output voltage $V_{m}$, and $V_{c}$ is the drive voltage, both are measurable quantities, so the control gain of the digital potentiometer can be updated through the following equation:

(10) $$e(n+1)=e(n)-cV_{m}V_{c}\;$$

A smaller step size is chosen that makes the gain converge to the global optimum. The algorithm is implemented in a self-sensing piezoelectric driving circuit by controlling the digital potentiometers with a microcontroller. The input voltage of the digital potentiometer is $\frac{C_{p}^{'}}{C_{1}+C_{p}^{'}}V$, which is driven by the capacitor divider, and the gain of the digital potentiometer is programmed by the microprocessor to drive the tap to the calculated value in each period, and the output voltage is $\left|e(n)\frac{C_{p}^{'}}{C_{1}+C_{p}^{'}}V\right|$. Meanwhile, the microcontroller controls the switch between the voltage follower and subtracts the circuit. When the value is between −1 and 0, the analog switching is connected with the voltage follower, and when the value is between 0 and 1, the analog switching is connected with the subtract circuit.

### 2.4. Adaptive Compensatory Mechanism for Local Sensing

The local strain problem of a piezoelectric self-sensing circuit has been discussed in the previous literature. In general, the measurement signal of self-sensing actuation is composed of the voltage induced by the global vibration of the coupled structure and a local strain induced by the converse piezoelectric effect. Within the linearity range of the piezoelectric patch, the local strain of the near field is proportional to the driving voltage applied to the actuation. On the other hand, the strain induced by the global vibration can be expressed as the sum of modal deformation, which is proportional to the modal displacement. According to Equation (1), the driving strain of the piezoelectric patch is given by $\sigma_{s}=cd_{t}E$, and the strain induced by the global vibration is given in the modal space: $\sigma_{t}=\sum_{i=1}^{n}\varphi_{i}(\overset{-}{x},\overset{-}{y},\overset{-}{z})x_{i}$ where $x_{i}$ is the *i*th modal variable and $\varphi_{i}$ is the *i*th modal participation factor. Then the induced charge on the terminal of measurement is given by:

(11) $$Q=\underset{\Omega }{∭}d(\sigma_{s}+\sigma_{t})d\Omega =\underset{\Omega }{∭}cdd_{t}Ed\Omega +\sum_{i=1}^{n}\underset{\Omega }{∭}cd\varphi_{i}x_{i}d\Omega \;$$

where Ω is the integration field. It can be seen from Equation (11) that the voltage induced by the local strain is proportional to the driving voltage applied to the piezoelectric patch, and the voltage induced by the global vibration can also be given by the form of modal space. The transfer function from the driving voltage to the measurement voltage is obtained by the Laplace transformation:

(12) $$\frac{V_{m}(s)}{V_{c}(s)}=\sum_{i=1}^{n}\frac{\varphi_{i}\beta }{\varphi_{i}s^{2}+2\xi \omega_{ni}s+{\left(\omega_{ni}\right)}^{2}}+\alpha \varphi_{i}\;$$

where α and β are the gain coefficients of driving and measurement voltage, respectively. By converting the transform function to the frequency domain, the frequency response function, which involves the effect of local strain, is also expressed as the summation of modal deformation and feedforward voltage, and thus the local strain part can be compensated by the feedforward compensation method. Considering the frequency response function, which involves the local strain effect and feedforward compensation:

(13) $$H(j\omega )=\sum_{i=1}^{n}(\frac{\varphi_{i}\beta }{-\varphi_{i}\omega^{2}+j2\xi \omega_{ni}\omega +{\left(\omega_{ni}\right)}^{2}}+\alpha \varphi_{i})-\frac{C_{p}^{'}}{C_{1}+C_{p}^{'}}ek_{2}\;$$

Since the modal deformation section of the frequency response function is quite small with the range apart from the natural frequency and irrelevant to the global vibration induced by the driving voltage, the object function is determined by:

(14) $$E=\sum_{i=1}^{n}{\left|H(j\omega_{i})V_{c}(j\omega_{i})\right|}^{2}\;$$

where i=1,2……,n represents the n frequency point, apart from the neutral frequency of the structure. Then, the partial deviation is taken for the potentiometer gain of the objective function:

(15) $$\frac{\partial E}{\partial e}=2\frac{C_{p}^{'}}{C_{1}+C_{p}^{'}}k_{2}\sum_{i=1}^{n}\left|H(j\omega_{i})V_{c}^{2}(j\omega_{i})\right|\;$$

Then the steep descent iterative expression based on frequency domain is given by:

(16) $$e(n+1)=e(n)-c\sum_{i=1}^{n}\left|H(j\omega_{i})V_{c}^{2}(j\omega_{i})\right|\;$$

**Figure 5:**
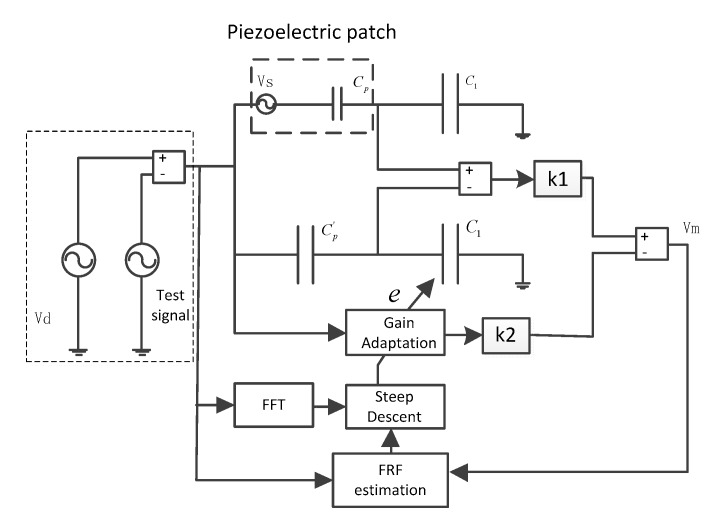
Schematic of the adaptive strategy for local strain.

The block diagram of the frequency domain steepest descent algorithm is shown in Figure 5; the driving voltage and measurement signal are acquired to estimate the frequency response function, and the measurement signal is transformed to the frequency domain by FFT (Fast Fourier Transformation). Then, the potentiometer gain is adapted by Equation (16).

### 2.5 Offline Adjustment Test

**Figure 6:**
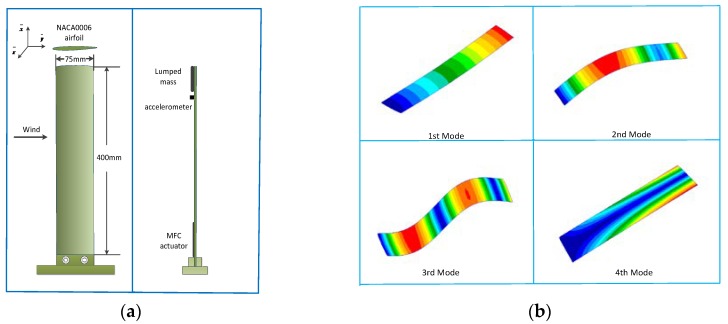
(**a**) Configuration of the flexible wing model; (**b**) mode shape of the flexible wing.

A wing model was designed to verify the control algorithm in a wind tunnel test. The wing model was made of elastic plastic with an elastic modulus of 1.1 Gpa, a density of 1430 kg/m^3^, and a Poisson ratio of 0.39. The cross-section of the wing model was NACA0006 and the max thickness was 6% of the chord length. A lumped mass of 36 grams was mounted on the tip of the wing as the counterweight in the wind tunnel test. Table 1 shows the modal parameter of the wing model. The wing root was vertically fixed on the base of the closed wind tunnel. The configuration of the actuator and sensor is shown in Figure 6a. MFC (Macro Fiber Composite) was adhered to the root of the wing model as the control actuator, and an accelerometer was mounted on the tip of the wing as the monitoring signal. Figure 6b shows the mode shape of the wing model.

The driving voltage of the piezoelectric material was the sinusoidal signal applied on an MFC through the power amplifier. The gain of the compensation tunnel was modified by the operational amplifier OPA552, which provided the voltage range of ±30 V to alleviate the feedforward interference of the measurement signal. The measurement voltage and driving voltage of the bridge were acquired by the AD module and the gain of the digital potentiometer voltage was adjusted in the STM32 microcontroller.

**Figure 7:**
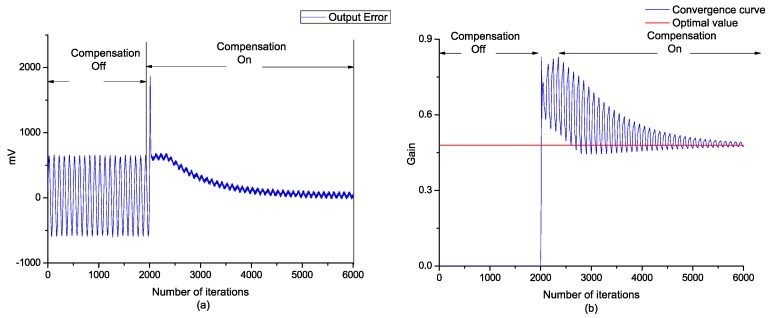
(**a**) Time domain steepest descent (TDSD) convergence curve of the measurement signal; (**b**) TDSD convergence curve of the positioner gain.

The offline sinusoidal driving voltage was determined by 2 V and 10 Hz, and the iterative step length of the steepest gradient was 0.0002. It can be seen that the final value of the digital potentiometer gradually converged to the ideal value after iteration, and the error curve tended to the minimum, as shown in Figure 7.

**Figure 8:**
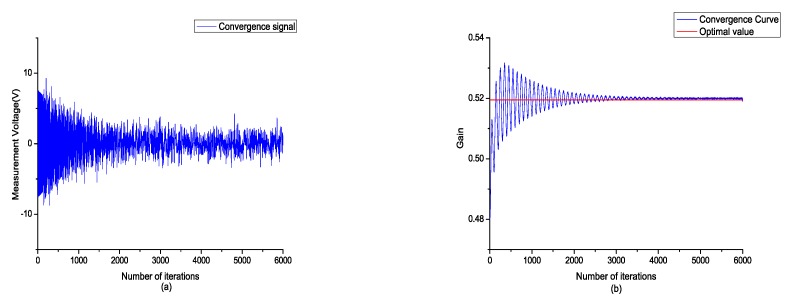
(**a**) Frequency domain steepest descent (FDSD) convergence signal of the measurement signal; (**b**) FDSD convergence curve of the positioner.

After the time domain steepest descent algorithm was implemented, the frequency domain steepest descent algorithm was utilized to alleviate the local strain. A bandpass signal within the range of 0 Hz to 50 Hz was applied to the piezoelectric electrode as a test signal, and the summation of the frequency response function (FRF) magnitude within the range from 35 Hz to 45 Hz was selected to be the object function of the frequency domain steepest descent algorithm. The output error and convergence curve of the gain are shown in Figure 8.

**Figure 9:**
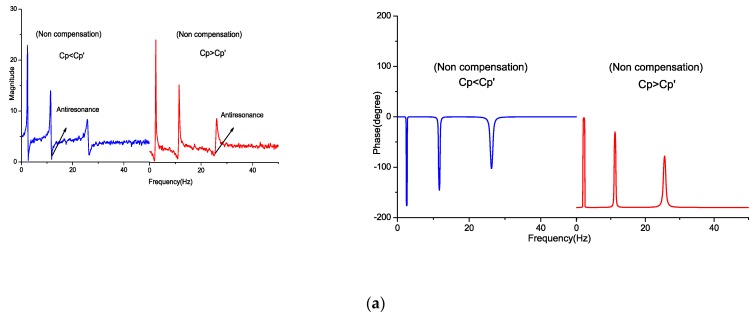

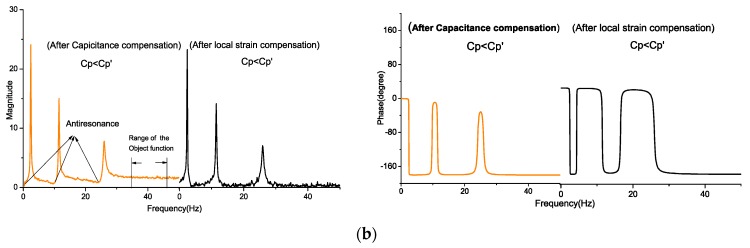
Frequency response function of bridge unbalance circuit (**a**) with no compensation, and (**b**) with feedforward compensation.

The frequency response function from the driving signal to the sensing signal was taken, which determined the balance of the bridge circuit and local strain. When the value of matching capacitance $C_{p}^{'}$ varied, the induced effect was reflected on FRFs. With the change in $C_{p}^{'}$, variation in the resonance and antiresonance locations of FRF can be observed. When the capacitance value of $C_{p}^{'}$ was less than $C_{p}$, the antiresonance occurred prior to the resonance, and the contrary occurred when $C_{p}^{'}$ was greater than $C_{p}$. Furthermore, since the driving voltage and local strain were added to the measurement signal, it can be observed that the magnitude of FRF moved upward compared to the matching capacitance. With the feedforward compensation, the antiresonance and magnitude bias were eliminated in FRF. Figure 9 shows FRF comparison with no compensation and after compensating.

## 3. Active Flutter Control Using Self-Sensing Circuit

### 3.1. Dynamic Equation of an Aeroelastic System

**Figure 10:**
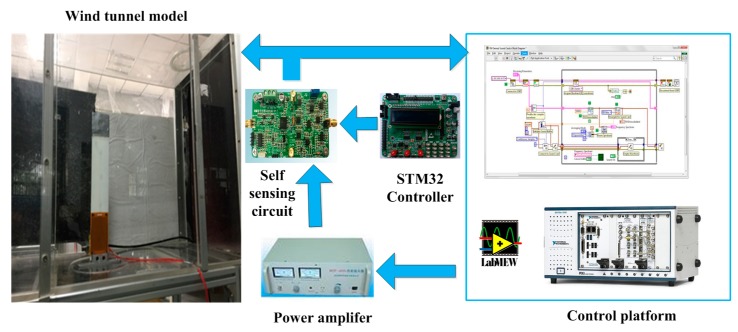
Experimental equipment of the wind tunnel test.

**Figure 11:**
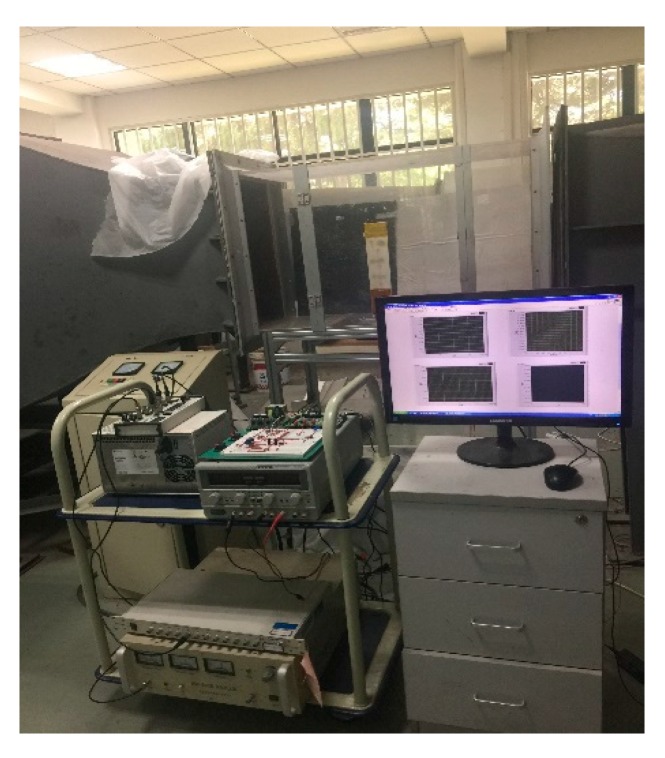
Scene of the wind tunnel test.

Flutter is a self-exiting aeroelastic phenomenon that occurs when fluid couples with energy in a structure motion, causing instability and excessive vibration. In this study, MFC and a self-sensing piezoelectric driving circuit were utilized to control the flutter of the flexible wing model. The aeroelastic equation is determined by the modal vibration formulation and unsteady aerodynamic force, which can be obtained as:

(17) $$M\overset{\cdot \cdot }{x^{'}}+C\overset{\cdot }{x^{'}}+Kx^{'}=F(u)+Q_{\xi \xi }(k_{r},M_{\infty })x^{'}\;$$

where M,C,K are the modal mass, modal damping, and modal stiffness matrix, respectively, $Q_{\xi \xi }(k_{r},M_{\infty })$ is the unsteady aerodynamic matrix under a modal coordinate, and F(u) is the dynamic system from the control output to the generalized force applied to the modal variable. The unsteady aeroelastic matrix changes with varying wind speed. When the wind speed exceeds the particular critical speed, the aeroelastic system turns unstable, and exhibits a strong vibration, so flutter control by MFCs and the self-sensing piezoelectric actuator will be introduced in the following part. Figure 10 shows the experimental equipment for the flutter suppression test and Figure 11 shows the scene of the wind tunnel test.

### 3.2. Experimental Setup of the Wind Tunnel Test

**Figure 12:**
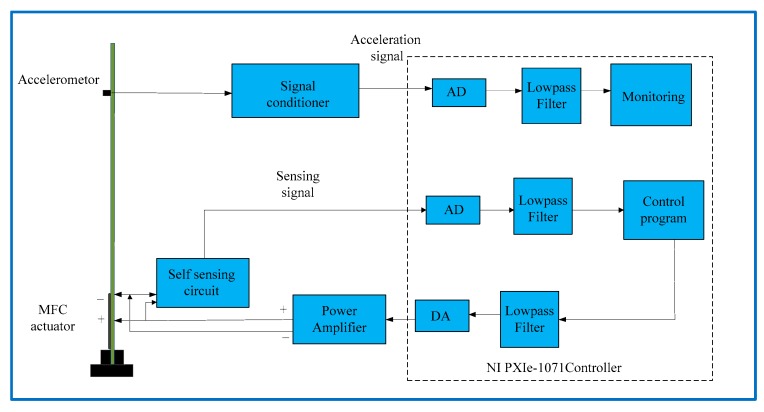
Block diagram of flutter control system of wing model for wind tunnel tests.

The control system configuration is shown in Figure 12. The self-sensing circuit was connected with an MFC to provide the driving voltage and obtain the sensing signal. The control and monitoring programmer was implemented in the embedded controller and programmed by LabVIEW. The control output signal was calculated and applied to the MFC actuator through a power amplifier. The vibration signal was acquired by the acceleration signal to monitor the performance of the control. The flutter controller was designed by the state-space model under wind speed 27 m/s, and sampling time was 0.002 s.

### 3.3. State-Space Model of Aeroelastic System

The aeroelastic system of the wind tunnel is modeled by the state-space equation:

(18) $$\begin{array}{l} \overset{\cdot }{x}=Ax+Bu\\ y=Cx+Du \end{array}$$

where A,B,C,D are the state-space matrix of the aeroelastic system, x is the state-space variable, y is the output, which is the signal measured by the MFC in the actual system, and u is the control signal, which is exported from the DA of the controller. Consider a dynamic feedback controller, which can be expressed as:

(19) $$\begin{array}{l} {\overset{\cdot }{x}}_{k}=A_{k}x_{k}+B_{k}y\\ u=C_{k}x_{k}+D_{k}y \end{array}$$

Substituting Equation (13) into Equation (12), the closed-loop system can be expressed as:

(20) $$\overset{\cdot }{x}=A_{cl}x\;$$

The closed-loop system of the uncertainty model can be expressed as:

(21) $$A_{c1}=\left[\begin{array}{cc} A+B_{}D_{K}C_{} & B_{2}C_{K}\\ B_{K}C_{} & A_{K} \end{array}\right]\;$$

The purpose of the controller design is to place the poles of the uncertain closed-loop system within a specific region. Prior to designing the controller, the state-space model of the aeroelastic system was determined by system identification. Frequency domain subspace identification was used to obtain the state-space model. Since flutter mode plays a dominant role in vibration, a two-order model was used to approximate the unstable mode. The identification algorithm was implemented based on the frequency response data to estimate the system matrix of the state-space model, and it exhibited reliable performance.

### 3.4. Pole Placement Controller Design

Since flutter is caused by instability of the aeroelastic system, and the poles of the system should be located in the left half of the complex plane for linear stability, pole placement controllers were designed to make closed-loop system poles in the left half of the complex plane beyond the flutter speed. The relevant content can be found in the literature [39,40]. In general, the pole placement controller makes the aeroelastic system stable, increasing the damping of the system and the anti-interference of the aeroelastic system to vibration excitation.

The feedback pole placement controller is available such that all of the poles of the closed-loop system (21) are located in the designated field if and only if two symmetric matrices R, S and state-space matrices $\bar{A_{k}},\bar{B_{k}},\bar{C_{k}},\bar{D_{k}}$ exist via:

(22) $$\Lambda (R,S)=\left[\begin{array}{cc} R & I\\ I & S \end{array}\right]>0\;$$

(23) $$\left[\begin{array}{ccc} L⊗\Lambda (R,S)+M⊗\Phi_{A}M^{T}⊗\Phi_{A}^{T} & * & *\\ M_{1}⊗\Phi_{B}^{T} & 0 & *\\ M_{2}⊗\Phi_{C} & I⊗\Phi_{D} & 0 \end{array}\right]\;$$

where

$\begin{array}{l} \Phi_{A}=\left[\begin{array}{cc} AR+B_{}\overset{-}{C_{K}} & A+B_{}D_{K}C_{}\\ \overset{-}{A_{K}} & SA+\overset{-}{B_{K}}C_{} \end{array}\right],\Phi_{B}=\left[\begin{array}{c} B_{}\\ SB_{} \end{array}\right]\\ \Phi_{C}=\left[\begin{array}{cc} C_{}R & C_{} \end{array}\right],\Phi_{D}=D_{} \end{array}$
∗ denotes the symmetric of the matrix. If the inequality in Equation (23) can be solved, then the state-space matrix of the controller is given by:
  $$\begin{array}{l} D_{K}={\overset{-}{D}}_{K}\\ B_{K}=N^{-1}{\overset{-}{B}}_{K}-N^{-1}SB_{}D_{K}\\ C_{K}={\overset{-}{C}}_{K}{\left(M^{T}\right)}^{-1}-D_{K}C_{2}R{\left(M^{T}\right)}^{-1}\\ A_{K}=N^{-1}{\overset{-}{A}}_{K}{\left(M^{T}\right)}^{-1}-B_{K}C_{}R{\left(M^{T}\right)}^{-1}-N^{-1}SB_{}C_{K}-N^{-1}S(A+B_{}D_{K}C_{})R{\left(M^{T}\right)}^{-1} \end{array}$$

As for the parameters of LMI in Equation (23), L,M present the pole location of the closed-loop system to be placed. In this study; L is set at 1.5, and M is set at 1; this means that the pole location of the closed loop system is located in the region where the character inequality is $L+z+\bar{z}<0$, The location of the pole indicates that all of the time constants of the system are greater than L/2=0.75, and the vibration signal will exponentially decay faster than $e^{-0.75t}$.

## 4. Results and Discussion

The wind tunnel experiment for validating the function of the proposed self-sensing method was designed as follows. First, system identification was implemented to obtain the state-space model of the aeroelastic system after the sensing signal was compensated. Second, the controller was designed based on the identified aeroelastic system by LMI. During the design, the controller was utilized for flutter suppression, and an adaptive strategy under the condition of capacitance mismatching was implemented in the wind tunnel test to compensate for the sensing signal. Finally, the same controller was applied to flutter suppression by self-sensing actuation with no compensation, and $C_{p}^{'}$ was not equivalent to $C_{p}$ for comparison.

Prior to the design of the controller, the aeroelastic model was identified by the frequency domain subspace method. As the critical speed of flutter is 27 m/s, the aeroelastic model under the critical flutter wind speed was identified. A bandpass signal that evolved the frequency of flutter was used as the excitation, and the aeroelastic system was identified under a wind speed of slightly less than 27 m/s. Then, the output signal of the self-sensing piezoelectric drive circuit was acquired. The frequency response function of input and output data was calculated by the correlation function for frequency state-space identification. The identified state space models under wind speed below 27 m/s is:

$$\begin{array}{l} A_{}=\left[\begin{array}{cc} -3.974 & -152.3\\ 256 & 0 \end{array}\right],B_{}=\left[\begin{array}{c} 2\\ 0 \end{array}\right]\\ C_{}=\left[\begin{array}{cc} 0.636 & 1.709 \end{array}\right],D_{}=0 \end{array}$$

Substituting the identified model into the linear matrix inequality (Equations (22) and (23)), the pole placement controller is given by:

$$\begin{array}{l} A_{k}=\left[\begin{array}{cc} -42.0951 & 188.1113\\ -200.1712 & 22.1701 \end{array}\right],B_{k}=\left[\begin{array}{c} 0.1791\\ -0.1012 \end{array}\right]\\ C_{k}=\left[\begin{array}{cc} -0.1682 & -0.1674 \end{array}\right],D_{k}=0 \end{array}$$

Before the flutter suppression experiment, the designed controller should be discretized. A zero-order hold transformation was used to convert the analog controller to the form of the difference equation. The discrete transfer function of designed controller is given by:

$$K(z)=\frac{-2.46\times 10^{-6}+2.003\times 10^{-5}z^{-1}+2.249\times 10^{-5}z^{-2}}{1-1.823z^{-1}+0.9623z^{-2}}$$

**Figure 13:**
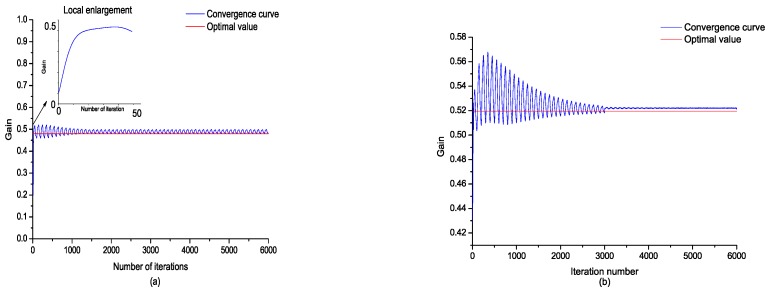
(**a**) TSDS convergence curve of online iteration; (**b**) frequency domain steepest descent (FDSD) convergence curve of online iteration.

The performance of the online adaption of potentiometer gain by TDSD and FDSD was validated. When flutter happens, the self-adaptive regulating function of the self-sensing piezoelectric circuit is opened. TDSD was first implemented to compensate the capacitance mismatching under the condition that the matching capacitance is less than the piezoelectric capacitance. After the gain of TDSD converged to the optimal value, the FDSD algorithm was implemented to alleviate local sensing. The convergence curve of potentiometer gain of online iteration is shown in Figure 13.

**Figure 14:**
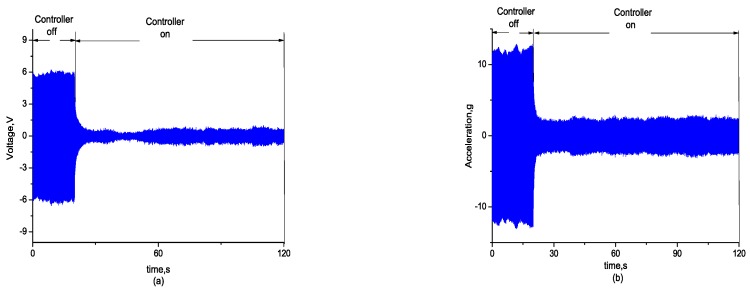
(**a**) Convergence signal of self-sensing measurement; (**b**) convergence signal of acceleration.

**Figure 15:**
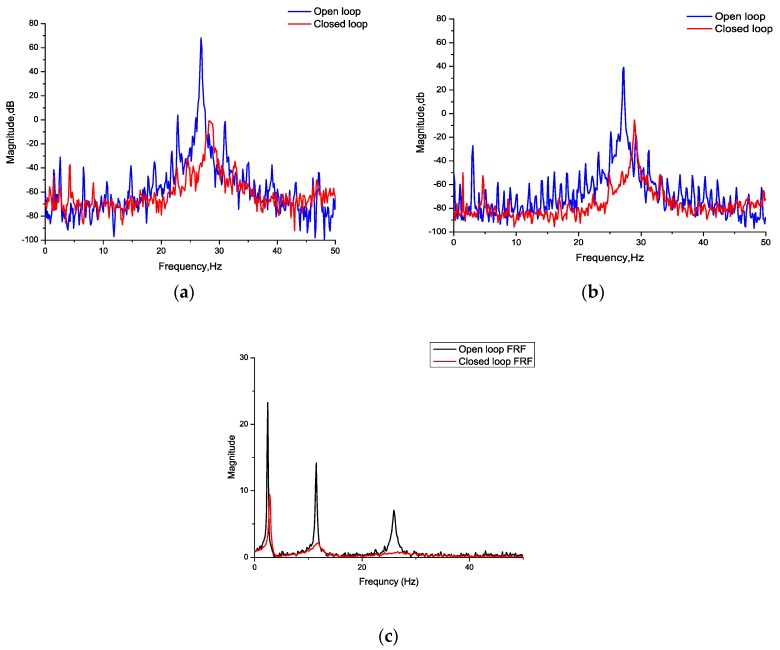
(**a**) Power spectrum of the self-sensing signal; (**b**) power spectrum of the acceleration signal; (**c**) comparison between the frequency response function (FRF) of the open loop and the closed loop.

Then, the effectiveness of the self-sensing compensation method in active vibration control was verified. While the wind speed was adjusted to the critical speed of the flutter at 27 m/s, the flexible wing swiftly turned from static state to vibration and reached a strong vibration state. After the controller was opened, the vibration signal and stress signal measured by the self-sensing piezoelectric actuator converged simultaneously, as shown in Figure 14 and Figure 15 shows the comparison between the open loop and closed loop FRF.

It can be seen in the comparison of the power spectrum that the peak value of the spectrum is significantly decreased. Since flutter is caused by the instability of the aeroelastic system, the critical frequency can be calculated from the eigenvalues of the state-space model of the aeroelastic system. When the controller is off, the eigenvalues of the aeroelastic system are natural, but when the controller is on, the pole placement controller is applied to the aeroelastic system, and a new closed-loop system is constructed. It is obvious that the eigenvalues of the original system and closed loop system are different, and the flutter frequency varies after the controller is on.

**Figure 16:**
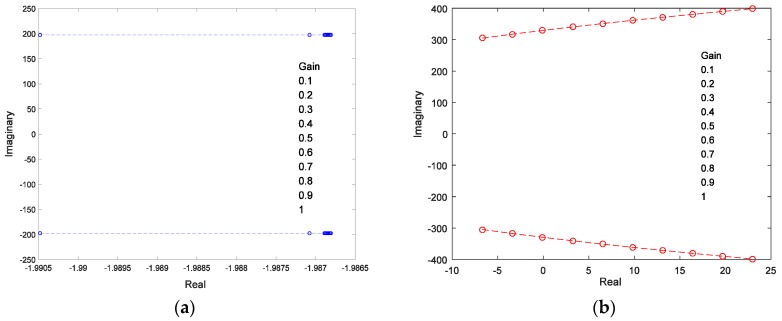
Root locus of closed loop system vs. feedforward gain. (**a**) Root locus of conjugated pole 1; (**b**) Root locus of conjugated pole 2

The root locus diagram of the eigenvalue of the system varies with the gain of the feedthrough inference, as shown in Figure 16. As the gain of inference is increased, one conjunction pole would pass through the imaginary axis, and the closed loop system is transformed to instablility.

**Figure 17:**
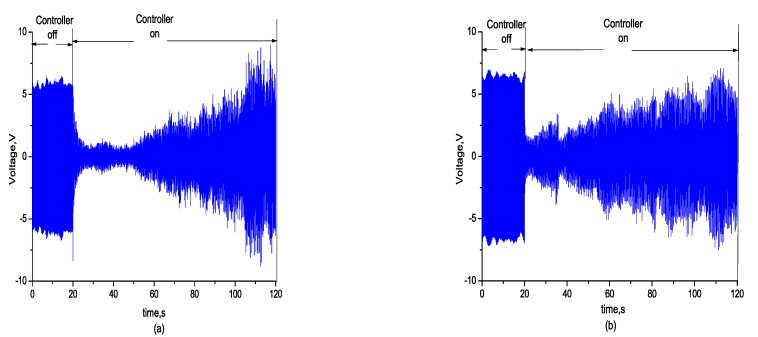
(**a**) Self-sensing signal vs. $C_{p}^{'}$ = 12 nF; (**b**) self-sensing signal with no local strain compensation.

To validate the effect of control performance under the condition with capacitance mismatch and local sensing, the control effects were compared under the capacitance mismatching conditions of 12 nf, with no compensation for local strain. Results shown in Figure 17 indicate that the feedforward gain led to the instability of the closed-loop system and the flutter signal diverged.

## 5. Conclusions

A new method for self-sensing actuation is proposed for the imbalance of bridge and local strain. The advantages of the new configuration are that the electric bridge circuit can be insured against capacitance mismatching and local sensing, and the induced feedforward interference of driving voltage in the measurement signal can be eliminated by compensation. The adaptive gain adjustment strategy can be utilized under the offline or online condition in the time domain and frequency domain that converges to the optimal value, which does not affect the application of the driving voltage. The proposed self-sensing piezoelectric driving circuit is applied to the flutter control of flexible wings by the controller that was designed by the pole placement algorithm. Theoretical analysis and experiment results show that the feedforward inference will greatly affect the stability and performance of the control and make the pole assignment fail. The proposed self-sensing piezoelectric method, which alleviates the feedforward interference, can avoid the influence of the bridge imbalance on the pole placement control for flutter suppression and is also applicable to other fields for active vibration control.

## Figures and Tables

**Table 1:** Comparison of numerical and experimental natural frequencies.

Mode	Numerical	Experimental	Mode shape
1	1.99 Hz	2.42 Hz	First bending
2	8.60 Hz	11.46 Hz	Second bending
3	23.47 Hz	25.93 Hz	Third bending
4	59.24 Hz	67.11 Hz	First torsion
